# An Adolescent with a Rare *De Novo* Distal Trisomy 6p and Distal Monosomy 6q Chromosomal Combination

**DOI:** 10.1155/2020/8857628

**Published:** 2020-08-31

**Authors:** Leia A. Peterman, Gail H. Vance, Erin E. Conboy, Katelynn Anderson, David D. Weaver

**Affiliations:** Department of Medical and Molecular Genetics, Indiana University School of Medicine, Indianapolis, IN, USA

## Abstract

We report on a 12-year-old female with both a partial duplication and deletion involving chromosome 6. The duplication involves 6p25.3p24.3 (7.585 Mb) while the deletion includes 6q27q27 (6.244 Mb). This chromosomal abnormality is also described as distal trisomy 6p and distal monosomy 6q. The patient has a Chiari II malformation, hydrocephalus, agenesis of the corpus callosum, microcephaly, bilateral renal duplicated collecting system, scoliosis, and myelomeningocele associated with a neurogenic bladder and bladder reflux. Additional features have included seizures, feeding dysfunction, failure to thrive, sleep apnea, global developmental delay, intellectual disability, and absent speech. To our knowledge, our report is just the sixth case in the literature with concomitant distal 6p duplication and distal 6q deletion. Although a majority of chromosomal duplication-deletion cases have resulted from a parental pericentric inversion, the parents of our case have normal chromosomes. This is the first reported *de novo* case of distal 6p duplication and distal 6q deletion. Alternate explanations for the origin of the patient's chromosome abnormalities include parental gonadal mosaicism, nonallelic homologous recombination, or potentially intrachromosomal transposition of the telomeres of chromosome 6. Nonpaternity was considered but ruled out by whole exome sequencing analysis.

## 1. Introduction

Distal trisomy 6p is a rare chromosomal disorder with a prevalence of less than one in a million. There have been approximately 40 reported cases [1] (decipher.sanger.ac.uk). The associated phenotypic findings are variable but generally include microcephaly, prominent forehead, anterior-chamber eye defects, glaucoma, narrow and short palpebral fissures, ptosis, ocular hypotelorism with a prominent nasal bridge, short and bulbous nose, microstomia with thin lips, low-set ears, ear anomalies, congenital heart defects, glomerulopathy, and kidney and urinary tract anomalies [1–4]. Other findings include failure to thrive, growth deficiency, developmental delay, speech delay, and intellectual disability [1–4].

Distal 6q deletions are also rare chromosomal disorders. The prevalence and incidence are unknown. However, there have been approximately 50 reported cases with the deletions ranging from 6q26 and 6q27 [5] (decipher.sanger.ac.uk). Individuals with terminal 6q deletion have variable features, which may include corpus callosal defects, hydrocephalus, periventricular nodular heterotopia (PNH), polymicrogyria, cerebellar anomalies, colpocephaly, microcephaly, retinal anomalies, epicanthal folds, ear anomalies, short neck, and spinal cord and vertebral anomalies [5–8]. Other findings may include seizures, feeding difficulties, speech delay, intellectual disability, growth deficiency, and hypotonia [5–8].

There is an even greater paucity of information with concomitant distal 6p duplication and distal 6q deletion, also called distal trisomy 6p and distal monosomy 6q. When distal trisomy 6p and distal monosomy 6q do occur together, the abnormalities normally are the result of a parental pericentric inversion. Here, we present a 12-year-old female with an apparent *de novo* distal trisomy 6p and distal monosomy 6q, associated with severe intellectual disability, and multiple central nervous and other system abnormalities.

## 2. Case Presentation

**Figure 1:**
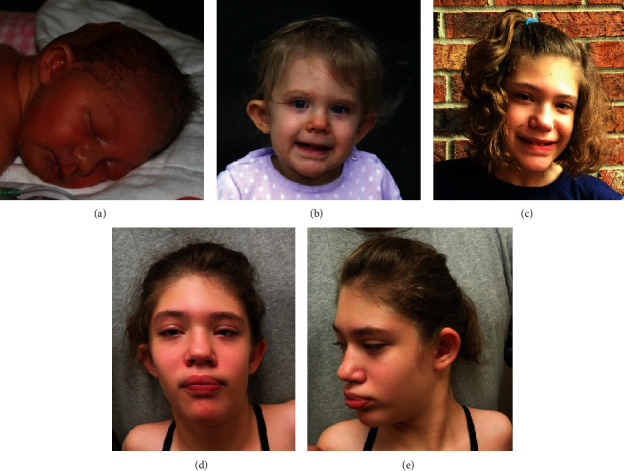
(a) Patient at 2 days of age. Observe the physical findings of prominent ear, crescent-shaped chin crease, and prominent philtrum. (b) Patient at 19 months of age. Note the strabismus, low-set and prominent ears, and crescent-shaped chin crease. (c–e) Patient at 11 years of age. Note the prominent ears, hypoplastic antihelix, narrow forehead, prominent and coarsened lips, widely spaced teeth, crescent crease of chin, and mild facial asymmetry (the right eyebrow is located higher than the left).

The patient is now a 12-year-old female who was diagnosed with a chromosomal abnormality at age 7. She is shown at various ages in Figures 1(a)–1(e). The patient has a history of Chiari II malformation, hydrocephalus, agenesis of the corpus callosum, microcephaly, bilateral renal duplicated collecting system, scoliosis, and myelomeningocele associated with a neurogenic bladder and bladder reflux. Other features have included seizures, feeding dysfunction, failure to thrive, sleep apnea, global developmental delays, intellectual disability, and absent speech.

During the pregnancy, drug exposures were limited to progesterone used to reduce the risk of miscarriage and heparin used to treat the mother's lupus anticoagulant syndrome. Fetal activity was normal. The patient's myelomeningocele was diagnosed by prenatal ultrasound at 22-week gestation. Because of the anomaly, she was delivered by cesarean section at 36-week gestation.

Her birth weight was 2.27 kg (25%ile), and her length was 45.7 cm (20%ile). Following birth, she was admitted to the neonatal intensive care unit for ten days for recovery from her myelomeningocele repair and ventriculo-abdominal shunt placement. During this period, she developed mild jaundice that required phototherapy. Other surgeries since this hospitalization have included Chiari II decompression, vesicostomy, sacral abscess drainage, G-tube placement, tonsillectomy, adenoidectomy, and scoliosis repair. Additional evaluations included normal metabolic testing including a lactate, pyruvic acid, serum amino acids, carnitine, and acylcarnitine profile.

At 7 years of age, a chromosomal microarray (CMA) and fluorescence *in situ* hybridization (FISH) analysis showed that she had a 6p25.3p24.3 (7.585 Mb) duplication and a 6q27q27 (6.244 Mb) deletion. At that time, she was able to walk using crutches but had no verbal communication. She also had a history of sleep apnea. Physical findings included prominent epicanthal folding bilaterally, strabismus, low-set ears with flattening of the outer helix, thickened lips, widely spaced but normally shaped teeth, and levoscoliosis.

Her most recent evaluation by us was at 11 years of age. She could walk unassisted for short distances but otherwise used a wheelchair. She was social and pointed to communicate her wants and needs; however, only nonspecific verbalizations were used without the use of words. She has continued to have recurrent gastrointestinal illnesses of unknown etiology. We found the same previously noted physical findings with the addition of hyperopia, striae over the breasts, pubic hair, and scoliosis. Levetiracetam and levocarnitine control her seizures, and she has been seizure free for over three years. Echocardiogram and electrocardiogram have been normal. Trio-based whole exome sequencing (WES) and mitochondrial DNA sequencing were performed including proband and parental samples, and all of which were reported negative. Although the duplication and deletion found on CMA was also identified on WES.

Our patient is one of four full siblings. Osteogenesis imperfecta (OI) type 1 is present in two siblings, the mother, and a maternal grandmother. The patient tested negative for OI type 1. The patient's mother has had her third recurrence of triple receptor negative breast cancer. Additionally, the mother has had two miscarriages thought to be related to her lupus anticoagulant syndrome. There is no additional family history of breast cancer or lupus anticoagulant syndrome. The patient is Caucasian with some Polish ancestry. Consanguinity and nonpaternity were denied.

## 3. Methods

### 3.1. Chromosome Analysis

GTG-banded metaphases from peripheral blood chromosome cultures treated with ethidium bromide were analyzed from the proband, her mother, and father following standard procedures. The proband's karyotype was reported as normal, 46,XX, at an average band level of 640 bands. The parents also had normal karyotypes, 46,XX, and 46,XY, at band levels of 626 and 616, respectively. A montage of chromosome 6 homologs was created and analyzed for each family member and was interpreted as normal.

### 3.2. Chromosome Microarray Analysis

Genomic DNA was extracted from whole blood. CMA was performed on the patient's DNA using the Applied Biosystems CytoScan HD array (Thermo Fisher, Scientific Carlsbad, CA) consisting of 1.2 million copy number and approximately 750,000 SNP oligonucleotide probes. The data analysis was performed using Chromosome Analysis Suite (ChAS) with the following filtering criteria: deletions ≥25 kb (a minimum of 25 probes) and duplications ≥50 kb (a minimum of 50 probes). The results were analyzed and reported using the NCBI human genome build 37.1 (GRCh37/hg19).

### 3.3. Fluorescence *In Situ* Hybridization

Array results were confirmed by fluorescence *in situ* hybridization (FISH) according to standard protocols. Ten metaphase spreads were analyzed using probes RP11-960L13 (6p25.1), RP11-205J13 (6p25.3), and RP11-755M8 (6q27) obtained from Empire Genomics (Buffalo, NY). The combination of dual-colored probes for 6p (red) and 6q (green) allowed for dual color localization on the derivative (6) and all normal 6 homologs.

### 3.4. Whole Exome Sequencing and Mitochondrial DNA (mtDNA) Analysis

Nuclear and mitochondrial genomic DNA of the proband was analyzed using next-generation sequencing (XomeDxPlus (trio), GeneDx Laboratories Inc., Gaithersburg, MD). WES was analyzed and reported using genome build GRCh37/UCSC hg19. Samples from the proband's mother and father were submitted as a trio.

The project was approved by the Indiana University School of Medicine Institutional Review Board (IRB #1011003014). Written consent was obtained from the patient's parents for participation in this research project.

## 4. Results

**Figure 2:**
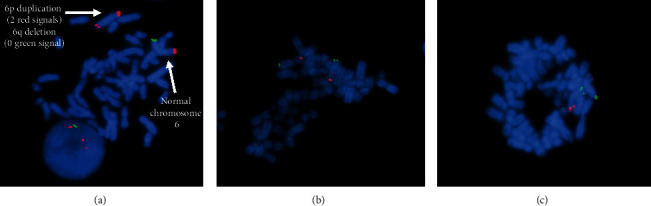
Fluorescence *in situ* hybridization results. (a) Patient. (b) Mother. (c) Father.

CMA results for our patient showed that she has 6p25.3p24.3 (7.585 Mb) duplication and a 6q27q27 (6.244 Mb) deletion (arr[hg19] 6p25.3p24.3(156,974-7,742,346)x3 and arr[hg19] 6q27(164,475,306-170,919,482)x1). These findings were confirmed by FISH (Figure 2(a)). Therefore, parental testing was done to determine whether either parent carried a pericentric inversion. The mother had nonclonal structural abnormalities on karyotype resulting from her chemotherapy. However, FISH studies for 6p and 6q were normal in the mother's sample (Figure 2(b)). The father's karyotype and FISH studies were normal (Figure 2(c)). Thus, neither parent carried a pericentric inversion. WES and mitochondrial genome sequencing and deletion analyses were performed on the patient. Samples from both the mother and father were also sent, and the WES analyses were performed as trio. The duplication and deletion found on CMA was also identified on WES, and paternity was confirmed. A comparison of variants between the mother, father, and proband suggested that the 6q deletion was of paternal origin. However, the origin of the 6p duplication could not be conclusively determined. The results for WES and mitochondrial genome sequencing and deletion analysis were otherwise negative.

## 5. Discussion

The 12-year-old female we presented here has a rare chromosomal structural abnormality that includes a distal 6p duplication or distal 6q deletion. Her physical abnormalities include Chiari II malformation, hydrocephalus, agenesis of the corpus callosum, microcephaly, bilateral renal duplicated collecting system, scoliosis, and myelomeningocele associated with a neurogenic bladder and bladder reflux. She also has a history of seizures, feeding dysfunction, failure to thrive, sleep apnea, global developmental delays, intellectual disability, and absence of verbal communication.

The common features of patients with distal trisomy 6p, distal monosomy 6q, and both distal trisomy 6p and monosomy 6q are presented in Table 1. Features associated with distal trisomy 6p more commonly include facial dysmorphisms, growth deficiency, developmental delay, intellectual disability, and congenital cardiac, renal, and ocular defects [2]. Features commonly seen in distal monosomy 6q include intellectual disability, developmental delay, facial dysmorphisms, and central nervous system abnormalities [9].

To our knowledge, there are only five reported cases with concomitant distal 6p duplication and distal 6q deletion without additional numerical or structural abnormalities (Table 1) [10–12]. We report the oldest patient to date with these chromosome abnormalities. All six of the cases share many features with only distal trisomy 6p or distal monosomy 6q. Also, all six of these cases have had a form of congenital anomaly of the kidneys and urinary tract system. Of the five cases who survived past term (cases 1–5), four had low-set ears and other ear anomalies and all five had failure to thrive. In addition, four of the children at ages 2 years or older (cases 1, 2, 4, and 5) had developmental delay, and three of the four cases had no verbal communication. However, verbal communication was not commented on in case 2. Absence of verbal communication does not appear to be a feature seen frequently in isolated distal trisomy 6p or isolated distal monosomy 6q deletions and may be unique to the presence of the deletion-duplication chromosome anomaly.

**Figure 3:**
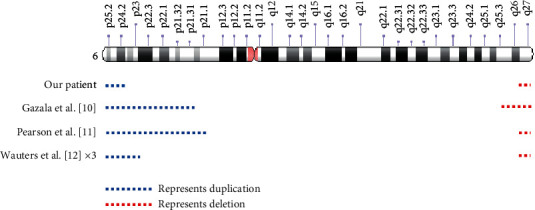
Idiogram comparison of distal duplication and deletion cases similar to our patient. Idiogram is from Genetics Home Reference https://ghr.nlm.nih.gove/chromosome/6#idiogram, credit: Genome Decoration Page/NCBI.

We report here the only such case with defined break points using CMA. Other reported cases identified the patient's chromosome abnormality by cytogenetic studies including either G-banded karyotype and/or FISH studies. When comparing the chromosome aberration of each case, the duplication and deletion sizes are variable (Figure 3). Our patient appears to have the smallest duplication size of all five cases, although it is difficult to compare the CMA breakpoints with G-banding in case 2.

The duplicated region in our patient, 6p25.3p24.3, includes 36 of Online Mendelian Inheritance in Man (OMIM) genes (Table 2), and the deleted region in our patient, 6q27q27, includes 25 OMIM genes (Table 3) (gena.tech). It is known that an imbalance in gene dosage may have deleterious effects with deletions generally resulting in a more severe clinical presentation than duplications [13]. There are several genes of interest within our patient's deleted region on 6q with associated phenotypic entries in OMIM (*PDE10A*, *TBXT*, *ERMARD*, *DLL1*, and *TBP*). The *TBXT* gene encodes for T protein, which is crucial for neurulation during embryonic development [14]. Additionally, the *DLL1* gene encodes a Notch ligand protein which is necessary for the Notch signaling cascade involved in neuronal differentiation and migration during the embryonic period [15]. Haploinsufficiency of one or both of these genes may have resulted in our patient's seizures, structural brain/spine abnormalities, and intellectual disability. Collection of additional patients with these gene deletions and associated functional studies will be necessary to further understand genotype-phenotype correlations.

When considering the mechanism leading to the presence of both distal trisomy 6p and distal monosomy 6q, a parental pericentric inversion is a likely possibility. Pericentric inversions can lead to the production of unbalanced gametes with duplications and deletions of the same chromosome distal to the inversion break points. This occurs as a result of the crossover of homologs within the inversion [16]. Neither of the parents of our case carries a detectable inversion as determined by normal G-banded karyotype and FISH studies, and paternity was confirmed via WES analysis. To the best of our knowledge, this is the first reported *de novo* case of distal trisomy 6p and distal monosomy 6q. However, we cannot rule out the possibility of gonadal mosaicism, where one parent may carry a pericentric inversion in his or her gametes. This latter possibility was suggested as a possible mechanism of a *de novo* duplication-deletion case of chromosome 10 [17]. Other reports in the literature propose the intrachromosomal transposition of subtelomeres as a predisposing mechanism for duplication and deletions as identified for chromosomes 1 and 2 [18]. During meiosis, a subtelomeric exchange occurs with the formation of a bouquet arrangement on the nuclear membrane, which, for our case, may have facilitated the exchange of distal 6p and 6q [18, 19]. Exchange of these subtelomeric regions occurring at the distal ends of chromosome 6 would not inhibit normal segregation if these small regions remained unpaired.

**Figure 4:**
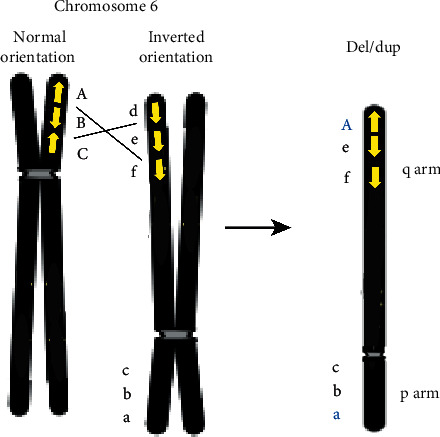
Proposed complex NAHR mechanism. The yellow arrows represent the LCRs, and the black lines indicate the possible exchange of two dissimilar regions of 6p and 6q that may carry an LCR in the same orientation. A crossover could lead from A to e to f,which would result in a duplication of A and a deletion of d. Image created and derived from reference [21].

We also considered that the chromosomal rearrangement was a result of recombination between segmental duplications on 6p and 6q of homologous chromosomes resulting in both a duplication and a deletion. Region-specific low-copy repeats (LCRs), also called segmental duplications, facilitate both inter- and intrachromosomal rearrangements via nonallelic homologous recombination (NAHR). This mechanism could explain our patient's *de novo* chromosome rearrangement, which has also been suggested for other chromosomal *de novo* duplication and deletion cases [19]. LCRs are segments of DNA containing greater than 90% of sequence identity, which constitute approximately 5% of our genome. LCRs are present across the entire human genome, and NAHR has been described as a frequent mechanism that underlies structural rearrangements of deletions and duplications [20, 21]. This latter mechanism is due to the homology of LCRs. LCRs on the same chromosome and in direct orientation have been shown to lead to duplication or deletion, which can occur in both meiotic and mitotic cells [22, 23]. However, in order to have both a deletion and duplication for our case, a much more complex NAHR mechanism would have had to occur. In the literature, there has been identification of a large LCR on 6p but not on 6q [24]. Thus, we propose that a complex mechanism may occur by a complete inversion of one of the chromosome 6 homologs and a crossover between 6p of one homolog and 6q of the other homolog due to an LCR in direct orientation (Figure 4) [21]. At this time, we are unable to conclusively determine the specific mechanism resulting in the 6p duplication and 6q deletion observed in our case.

## 6. Conclusions

In summary, we describe here a 12-year-old female with a rare karyotype including a 6p25.3p24.3 (7.585 Mb) duplication and a 6q27q27 (6.244 Mb) deletion. To our knowledge, there are only five other reported cases that are similar to ours, and many of these cases share common features associated with isolated trisomy 6p and isolated monosomy 6q. However, absent speech does not appear to be a common feature seen in either distal trisomy 6p or distal monosomy 6q, and may be unique to the presence of the deletion/duplication chromosome anomaly. Furthermore, what makes our case unique is that the origin of the chromosomal rearrangement is not due to an identifiable parental pericentric inversion as one would expect, but instead by an unknown alternate mechanism. Other patients will need to be identified and studied to determine underlying mechanisms leading to the aberration and the associated genotype-phenotype correlations.

## Figures and Tables

**Table 1:** Comparison of phenotypic features of patients with distal trisomy 6p and distal monosomy 6q.

Phenotypic features	Common features of distal trisomy 6p [1–4]	Common features of distal monosomy 6q [5–8]	Case 1: our patient 46,XX 6p25.3p24.3 dup; 6q27-qter del (12 y/o)	Case 2: Gazala et al. [10], patient 46,XX 6p21.2-pter dup; 6q25.3-qter del (4 y/o)	Case 3: Pearson et al. [11], patient 46,XX 6p21-pter dup; 6q27-qter del (died at 2 months old)	Case 4: Wauters et al. [12], patient 46,XX 6p23-pter dup; 6q27-qter del (2 y/o)	Case 5: Wauters et al. [12], patient 46,XY 6p23-pter dup; 6q27-qter del (8 y/o)	Case 6: Wauters et al. [12], patient 46,XY 6p23-pter dup; 6q27-qter del (elective termination at 13 weeks)
Abnormalities of the corpus callosum		+	+					
Other brain abnormalities		+	+		+		+	
Microcephaly	+		+	+		+		
Prominent forehead	+		−			+	+	
Anterior-chamber eye defects	+		−					
Retinal anomalies		+	−					
Glaucoma	+		−					
Narrow and short palpebral fissures	+		−					
Epicanthal folds		+	+			+		
Ptosis	+		−					
Ocular hypotelorism	+		−					
Other eye abnormalities			+		+	+	+	
Prominent nasal bridge	+		−					
Short bulbous nose	+		−		+			
Microstomia	+		−	+		+	+	
Thin lips	+		−			+	+	
Abnormal dentition			+	+				
Low-set ears	+		+	+		+	+	
Ear anomalies	+	+	+	+		+	+	
Short neck		+	−				+	
Congenital heart defects	+		−		+	−	−	
Glomerulopathy	+		−					
CAKUT	+		+	+	+	+	+	+
Spinal cord anomalies		+	+					
Vertebral anomalies		+	+	+				
Talipes equinovarus			−		+	+		
Hemangioma			−		+	+		
Growth deficiency	+	+	+			+	+	
IUGR			−	+			+	
Failure to thrive	+		+	+	+	+	+	
Developmental delay	+	+	+	+		+	+	
Intellectual disability	+	+	+			+	+	
Seizures		+	+				+	
Feeding difficulties		+	+			+	+	
Hypotonia		+	+					
Speech delay	+	+	−			−	−	
Absent speech			+			+	+	
Other features			a	b	c	d		

Note: a: recurrent gastrointestinal illnesses, striae of the breasts, and sleep apnea; b: triangular face; c: cranial synostosis; d: hypertonia and rectal prolapse. (+) indicates that feature is present. (−) indicates that feature is absent. Blank spaces indicate features were not reported as present or absent. IUGR: intrauterine growth restriction. CAKUT: congenital anomalies of the kidney and urinary tract. y/o: years old.

**Table 2:** 6p25.3p24.3 OMIM genes and OMIM ID number.

*DUSP22*/#616778	*SERPINB1*/130135	*WRNIP1*/608196	F13A1*/*134570
*IRF4*/#601900	*SERPINB9*/601799	*FAM50B*/614686	LY86*/*605241
*EXOC2*/615329	*SERPINB6*/173321	*PRPF4B*/602338	RREB1*/*602209
*HUS1B*/609713	*NQO2*/160998	*ECI2*/608024	SSR1*/*600868
*FOXQ1*/612788	*RIPK1*/603453	*CDYL*/603778	CAGE1*/*608304
*FOXF2*/603250	*BPHL*/603156	*RPP40*/606117	RIOK1*/*617753
*FOXCUT*/615976	*TUBB2A*/615101	*LYRM4*/613311	DSP*/*125647
*FOXC1*/601090	*TUBB2B*/612850	*FARS2*/611592	BMP6*/*112266
*GMDS/*602884	*PSMG4/*617550	*NRN1/*607409	*SLC22A23/*611697

Gene/OMIM ID.

**Table 3:** 6q27q27 OMIM genes and OMIM ID number.

*PDE10A* ^*∗*^/#610652	*CEP43*/605392	*TCP10L3*/187020	*C6orf120*/616987	FAM120B/612266
*TBXT* ^*∗*^/#601397	*CCR6*/601835	*AFDN*/159559	*PHF10*/613069	PSMB1/602017
*MPC1*/614738	*GPR31*/602043	*DACT2*/608966	*TCTE3*/186977	TBP^*∗*^*/*600075
*RPS6KA2*/601685	*HPAT5*/616837	*SMOC2*/607223	*ERMARD* ^*∗*^/615532	PDCD2/600866
*RNASET2/*612944	*UNC93A/*607995	*THBS2/*188061	*DLL1* ^*∗*^/606582	*KIF25/*603815

^*∗*^Associated with autosomal dominant disorders.

## Data Availability

No data were used to support the findings of this study.
